# Sex- and age-specific variation of gut microbiota in Brandt’s voles

**DOI:** 10.7717/peerj.11434

**Published:** 2021-06-08

**Authors:** Xiaoming Xu, Zhibin Zhang

**Affiliations:** 1 State Key Laboratory of Integrated Management of Pest Insects and Rodents, Institute of Zoology, Chinese Academy of Sciences, Beijing, Beijing, China; 2 CAS Center for Excellence in Biotic Interactions, University of Chinese Academy of Sciences, Beijing, Beijing, China

**Keywords:** Gut microbiota, Sex, Age, Wild animal

## Abstract

**Background:**

Gut microbiota plays a key role in the survival and reproduction of wild animals which rely on microbiota to break down plant compounds for nutrients. As compared to laboratory animals, wild animals face much more threat of environmental changes (e.g. food shortages and risk of infection). Therefore, studying the gut microbiota of wild animals can help us better understand the mechanisms animals use to adapt to their environment.

**Methods:**

We collected the feces of Brandt’s voles in the grassland, of three age groups (juvenile, adult and old), in both sexes. We studied the gut microbiota by 16S rRNA sequencing.

**Results:**

The main members of gut microbiota in Brandt’s voles were Firmicutes, Bacteroidetes and Proteobacteria. As voles get older, the proportion of Firmicutes increased gradually, and the proportion of Bacteroides decreased gradually. The diversity of the microbiota of juveniles is lower, seems like there is still a lot of space for colonization, and there are large variations in the composition of the microbiome between individuals. In adulthood, the gut microbiota tends to be stable, and the diversity is highest. In adult, the abundances of *Christensenellaceae* and *Peptococcus* of female were significantly higher than male voles.

**Conclusions:**

The gut microbiota of Brandt’s vole was influenced by sex and age, probably due to growth needs and hormone levels. Gut microbiota of wild animals were much influenced by their life-history reflected by their age and sex. Future studies will be directed to identify functions of these “wild microbiota” in regulating physiological or behavioral processes of wild animals in different life stage or sexes.

## Introduction

The intestinal microbiome maintains a commensal relationship with the intestinal mucosa of a healthy individual and plays an essential role of metabolism and immunity in the host (Bäckhed et al., 2005; Rup, 2012). Variations in the gut microbiome are often linked to age (Kato et al., 2017), host genes (Groussin et al., 2017; Sasson et al., 2017), lifestyle (De Filippo et al., 2010) and epigenetic changes (Li et al., 2018).

Studies showed the gut microbiota of animals would change significantly with aging (Claesson et al., 2012). The composition of the gut microbiota differs between infants, adults, and elders (Conlon & Bird, 2014; Ottman et al., 2012). For example, the diversity of gut microbiota in fecal samples of children was significantly lower than in adults (Yatsunenko et al., 2012). The change of gut microbiota with age can also reflect the growth trajectory of human life (Odamaki et al., 2016), since the differences of microbiota are affected by the diet and physiological status of different ages (Jeffery & O’Toole, 2013; Wu et al., 2011; David et al., 2014).

Sexes also affect the composition and proportion of gut microbiota Bolnick et al. (2014). The diversity of human gut microbiota was higher in females than in males after puberty (Sinha et al., 2019). Several studies have reported sex variation in the microbiome composition of mice (Markle et al., 2013; Kaliannan et al., 2018; Org et al., 2016; Yurkovetskiy et al., 2013). In human studies, women having higher alpha diversity than men (Sinha et al., 2019; De la Cuesta-Zuluaga et al., 2019). The effects of sex on gut microbiota are mainly regulated by sex hormones (Pace & Watnick, 2020), for example, estrogen stimulates IgA secretion to affect the gut microbiota.

Most studies have been carried out with humans or laboratory animal samples, while studies on wild animals are rare (Greyson-Gaito et al., 2020). There are many differences in physiological responses between wild animals and laboratory animals. Studies have shown that the alpha-diversity of the gut microbiota of wild deer mice is higher than that of captive deer mice; the difference of the microbiota is mainly reflected in the family level. *Ruminococcus*, *Laospirillum* and *Helicobacter* genera are the present in wild deer mice, and the marker bacteria for captive deer mice was Muribaculaceae (Schmidt, Mykytczuk & Schulte-Hostedde, 2019). Wild animals live shorter lifespan, they face more infections, though laboratory animals may face more stress due to limited space. Therefore, understanding the change of gut microbiota in wild animals would be much better to explain the effect of environment on host. In addition, it will allow us to uncover the mechanisms by which animals adapt to their natural environments.

Brandt’s vole (*Lasiopodomys brandtii*) is widely distributed in grasslands of Inner Mongolia, eastern Mongolia, and southeastern Russia’s outer Baikal. It is a typical social small herbivore species (Zhang & Wang, 1998). The lifespan of the wild Brandt’s vole is about 12 months. Overwintering voles often start to breed in May, and ends by August. They may breed two times in one breeding season. Most newborn voles in spring or summer do not take part in breeding in the current year due to high suppressing pressure of overwintering voles (Liu & Sun, 1993; Shi et al., 1999; Ren et al., 2016). The Brandt’s vole feeds on up to 33 species of plants, and it prefers *Leymus chinensis*, *Stipa krylovii*, and *Medicago varia* (Zhang & Wang, 1998; Zhou, Zhon & Wang, 1993). In the wild, studies have found that climate change can affect changes in Brandt’s vole populations through a perennial rhizomatous grass (*Leymus chinensis*) species -intestinal microbes (Li et al., 2020). Several studies on the gut microbes of laboratory Brandt’s voles have pointed at Firmicutes and Bacteroidetes phylums as the dominant microbiota in their guts (Li et al., 2020). The coprophagy, which contributes to stability of their gut microbiota, and functions of microbial metabolism, energy homeostasis, and cognitive abilities (Bo et al., 2020). The huddling behavior of Brandt’s vole could be beneficial to reducing inflammation-associated bacteria (Zhang et al., 2018), but under high housing density, disease- or stressful hormone-related bacteria of voles would increase (Liu et al., 2020).

The purpose of this study aims to examine the impacts of sex and aging on microbiotata of Brandt’s voles, and to discuss the difference of microbiotat between wild and laboratory voles, so as to to understand the ecological and evolutionary role of the gut microbiota throughout the lifetime of the voles in wild conditions.

## Materials & Methods

### Animal trapping

The animals were captured from the grasslands of East Ujim Qin Banner, Xilingol League area of Inner Mongolia (115^∘^58′E, 45^∘^33′N), China, in July 2019. The age of an animal was determined by its coat color, body weight, and reproductive organ status by referring to previous studies (Liu & Sun, 1993; Ren et al., 2016). First of all, we distinguished between overwintering voles and new-borne voles of the current year according to the color of the animal’s back. Overwintering voles are light gray-white, new-borne voles are yellow or yellow-brown, while young new-borne voles had a distinctive fluffy hair. Then, according to the body weight and reproductive status, we further distinguished the juvenile voles and adult ones (i.e., sexually mature voles). The body weight of overwintering and adult voles was between 35-45 g, while the weight of juvenile voles was less than 20 g. When two voles are similar in weight, the overwintering voles and adult voles can be further distinguished based on their coat color and reproduction status. Although adult voles are sexually mature and may have participated in reproduction, the overwintering voles, were the main breeding force during this period. For male, the testis position was used to identify the immature or mature voles, testis falling from abdominal cavity to scrotum indicates sexual maturity. In females, the state of the vaginal opening and the presence of vaginal plug are used to identify juvenile voles and adult voles in female adult voles. Based on these criteria, all captured voles were divided into three age groups: juveniles (female: *n* = 7, male: *n* = 7), adults (female: *n* = 8, male: *n* = 8) and olds (female: *n* = 8, male: *n* = 8). After review by the Experimental Animal Welfare Ethics Review Committee of the Institute of Zoology, Chinese Academy of Sciences (No. 2019FY100300), it is believed that all animal experiment operations meet the requirements of the state and this unit for the ethical review of laboratory animal welfare. 

### Sample collection

We placed a trap near the Brandt’s voles hole group. A piece of sterile paper was placed under the trap. The feces of the captured Brandt’s voles would remain on the paper. The trapping occurred on a plot (about 60 m × 80 m) in the grassland. The trap was placed at four o’clock in the afternoon, and the volels were caught about half an hour later. Number each vole and record the vole’s physical condition. Feces are collected within 5 minutes, and the number of fecal pellets collected for each animal is 3 to 5 pellets, and stored them at −80^∘^C.

### 16S rRNA sequencing and bioinformatics analysis

Total genome DNA from samples was extracted using the cetyltrimethylammonium bromide (CTAB) method, the purity and concentration of DNA were determined by Gel electrophoresis and diluted to 1 ng/ul by sterile water. To amplify the V4 (Caporaso et al., 2011) hypervariable region of the microbial 16S rRNA gene, the universal primers 515F (5′-GTGCCAGCMGCCGCGGTAA-3′) and 806R (5′-GGACTACHVGGGTWTCTAAT-3′) with 12 nt unique barcodes at 5′-end of 515F were used. The library was constructed with Thermofisher Ion Plus Fragment Library kit 48 rxns. After Qubit quantitative analysis and library test, the library was sequenced with Thermofisher Ion S5^TM^XL. The raw sequence data were processed using QIIME 1.9.1 (http://qiime.org/tutorials/tutorial.html) (Caporaso et al., 2010). By using Cutadapt (1.9.1), all sequences were trimmed and assigned to each sample based on their barcodes. All sequences were clustered into operational taxonomic units (OTUs) at a 97% identity threshold using Uparse (7.0.1001) (Li & Godzik, 2006). The Silva132 Database (Yilmaz et al., 2014) was used based on Mothur algorithm to annotate taxonomic information. OTU sequences were annotated, and the SSUrRNA databases of Silva132 were used for species annotation analysis (threshold: 0.8-1). The phylogenetic relationship of all OTU sequences can be obtained by comparing multiple sequences with MUSCLE (3.8.31) software, and the phylogenetic tree can be established. The alpha diversity, including Observed species, Shannon index, Simpson, Chao1 and Faith’s Phylogenetic diversity, were calculated with QIIME1.9.1 and displayed with R software (Version 2.15.3). The rarefaction depth is 47767. The unweighted and weighted UniFrac distance metrics, were used to calculate community similarity (beta diversity) (Lozupone & Knight, 2005) with the QIIME1.9.1.

### Statistical analysis

For alpha diversity, we used Kruskal-Wallis test. For beta diversity, the principal co-ordinates analysis (PCoA) was used to show the plot and the difference between groups were tested by “ANOSIM” (permutations = 999) in the R “vegan” package (Dixon, 2003). We calculated the relative abundance of microbial taxa at phylum level and analyzed them by Kruskal-Wallis. The linear discriminant analysis (LDA) Effect Size (LEfSe) method was used to assess differences in microbial communities using an LDA score threshold of 2 (Segata et al., 2011). Analysis of composition of microbiomes (ANCOM) in qiime2 (2019.10) was used to analyze relative abundance of OTU tables (Mandal et al., 2015).

## Results

### Gut microbial diversity

**Figure 1:**
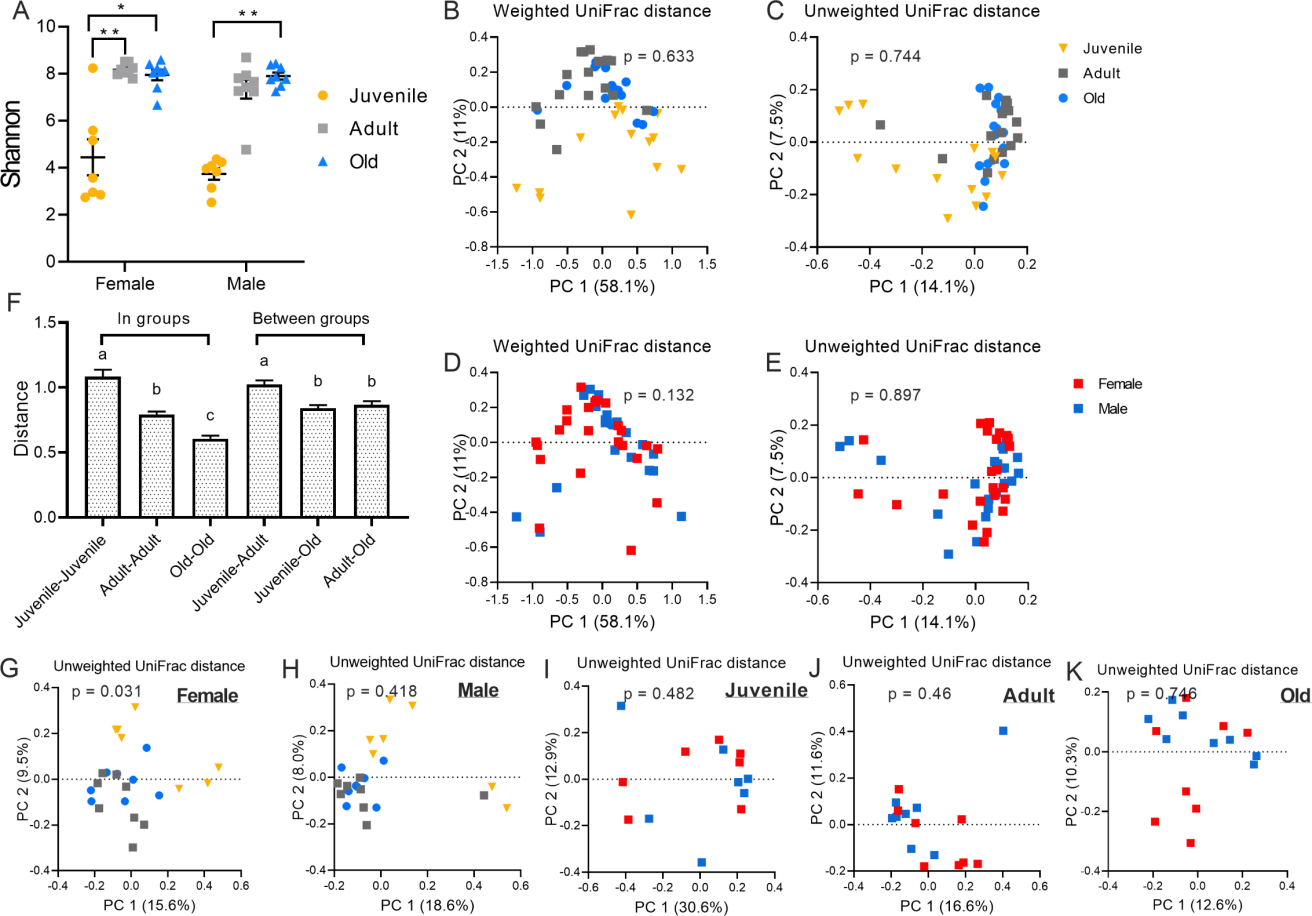
Variation of gut microbial diversity between age and sex groups. (A) Alpha diversity (Shannon index) of bacterial communities across groups (Kruskal-Wallis, *p < 0.05). (B) PCoA plot is based on weighted UniFrac distance metrics representing the differences in fecal microbial community structure in different groups. (C) PCoA plot is based on unweighted UniFrac distance metrics in different groups. (D) PCoA plot is based on weighted UniFrac distance metrics representing the differences in microbial community structure in different sex. (E) PCoA plot is based on unweighted UniFrac distance metrics representing the differences in microbial community structure in different sex. (F) Comparison of the weighted unifrac distances between groups and within groups. (G) PCoA plot is based on unweighted UniFrac distance metrics representing the differences in microbial community structure in different sex in juvenile, adult and old groups and differernt age in female and male groups (ANOSIM, *p < 0.05). Data are means ± SEM (Female-young: *n* = 7, Female-adult: *n* = 8, Female-old: *n* = 8; Male-juvenile: *n* = 7, Male-adult: *n* = 8, Male-old: *n* = 8).

From Kruskal-Wallis, Shannon index of gut microbiota showed significant difference between age groups (Fig. 1A, *p* < 0.001); it was significantly lower in juveniles than adult in female (*p* = 0.0048), also juvenile group was lower than old group in female (*p* = 0.029). In male, juvenile group was lower than old group (*p* = 0.034). From Table 1, the Observed species (*p* < 0.001), Simpson (*p* < 0.001), Chao1 (*p* < 0.001) and Faith’s Phylogenetic Diversity (*p* < 0.001) exhibited the same results: juvenile group had the lowest alpha diversity in both female and male, and there was no significant effect between adult and old. Observed species (Female: juvenile v.s. adult *p* = 0.01; juveniles v.s. old *p* = 0.012; Male: juvenile v.s. old *p* = 0.004). Simpson (Female: juvenile v.s. adult *p* = 0.015; Male: juvenile v.s. old *p* = 0.047). Chao1 (Female: juvenile v.s. adult *p* = 0.019; juvenile v.s. old *p* = 0.009; Male: juvenile v.s. old *p* = 0.008). Faith’s Phylogenetic Diversity (Female: juvenile v.s. adult *p* = 0.009; juvenile v.s. old *p* = 0.009; Male: juvenile v.s. old *p* = 0.006).

**Table 1:** Alpha diversity (5 indexes) of bacterial communities across groups (Kruskal-Wallis). The Observed species, Shannon, Simpson, Chao1 and Faith’s Phylogenetic Diversity in both female and male (juvenile v.s. adult; juveniles v.s. old; adult v.s. old) groups (Kruskal-Wallis).

		Observed_species	Shannon	Simpson	Chao1	Faith’s Phylogenetic Diversity
Female	Juvenile	875.7 ± 118.7	5.5 ± 0.69	0.90 ± 0.04	118.5 ± 1571.8	4.45 ± 81.7
	Adult	1,529.7 ± 32.3	8.2 ± 0.09	0.99 ± 0.001	1,655.2 ± 31.3	82.2 ± 1.07
	Old	1,508.5 ± 69.6	7.9 ± 0.2	0.98 ± 0.004	1,645.4 ± 73.2	82.7 ± 1.07
Male	Juvenile	879.3 ± 103.3	5.7 ± 0.6	0.92 ± 0.031	1,027.6 ± 113.8	58.1 ± 3.5
	Adult	1,425 ± 38.6	7.3 ± 0.4	0.96 ± 0.016	1,571.8 ± 38.9	81.7 ± 4.6
	Old	1,618 ± 120.9	7.9 ± 0.15	0.98 ± 0.002	1,810.6 ± 146.0	98.3 ± 9.5
*P* value	6 groups	<0.001	<0.001	<0.001	<0.001	<0.001

Beta diversity of gut microbiota based on Unweighted and weighted UniFrac distances revealed no significant differences between age groups (Figs. 1B, 1C) and sex groups (Figs. 1D, 1E). For both Weighted UniFrac distances and Unweighted UniFrac distances metrics, gut microbiota of adult group was more associated with the old group than to the juvenile group, and gut microbiota of both adult and old were less associated with the juvenile group. By comparing the weighted UniFrac distances between groups and within groups, we found that the intra-group difference was largest in young voles, while individuals of old group had a more similar microbiota structure (Fig. 1F). Comparing the distance between groups, we found that the greatest difference in the microbiota structure was between juvenile and adult voles (Fig. 1F, *F*_5,1028_ = 22.868, *p*  < 0.001). In female voles, there was significant difference between juvenile, adult and old groups (Figs. 1G–1K).

### Gut microbial composition

**Figure 2:**
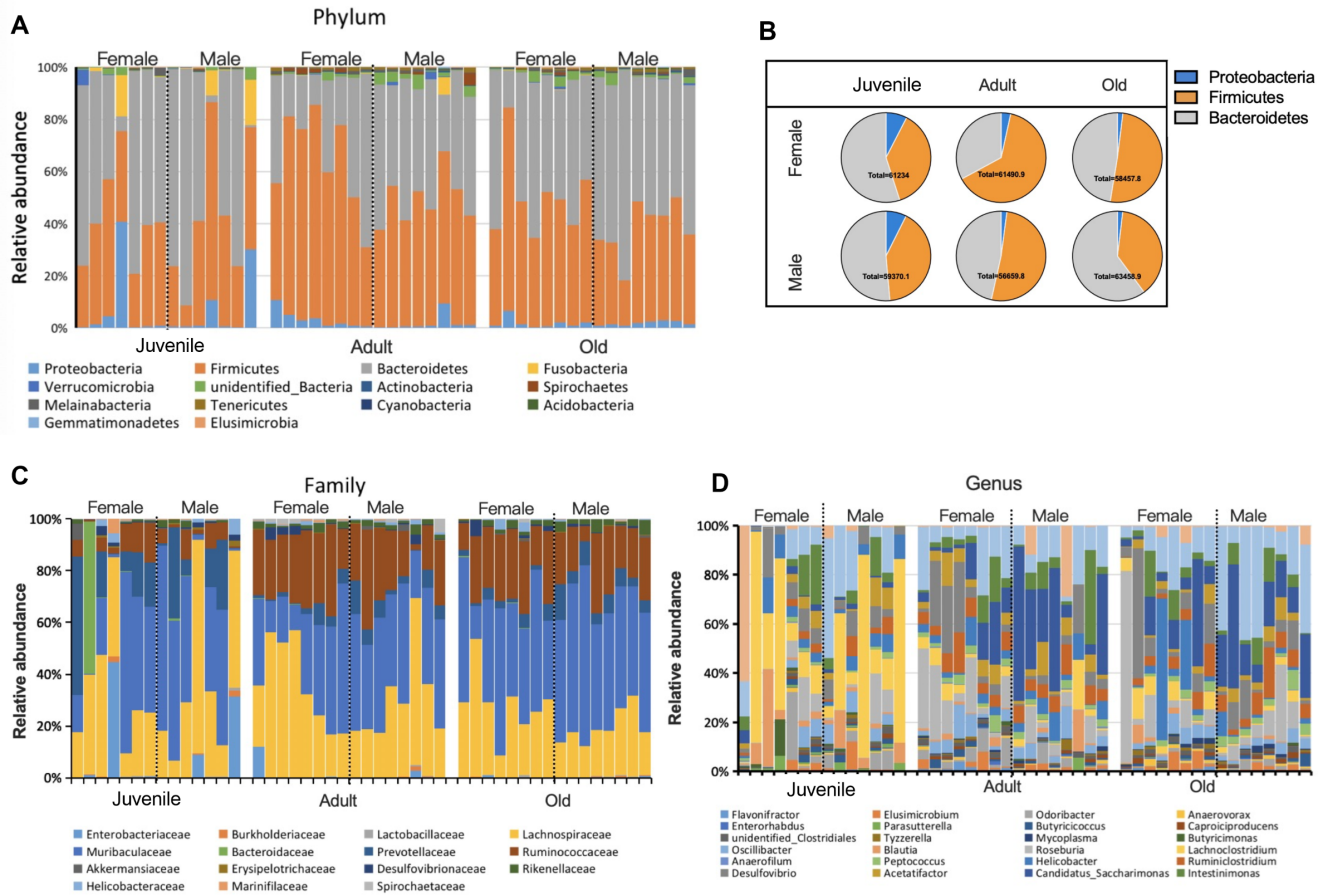
Variation of gut microbial composition between sex and age groups. (A) Abundance represented as the proportions of OTUs classified at the phylum rank. (B) Pie chart of three main phylum in different age and sex. (C) Abundance represented as the proportions of OTUs classified at the family rank. (D) Abundance represented as the proportions of OTUs classified at the genus rank.

From our results, total sequences were assigned to 35 phylum, 227 families, and 505 genera. The most abundant bacterial phylum were Firmicutes (mean = 43.98%), Bacteroidetes (47.36%), and Proteobacteria (3.52%). The composition of each sample can be visualized at the phylum level in Fig. 2A. The pie chart showed that there were sex differences in the changing trend of the three main phylum with increase of age. In adults, the proportion of Firmicutes in females was higher than that in males, and Bacteroides was lower in females than males, however, in old group, the opposite results were observed (Fig. 2B). The proportion of Firmicum and Bacteroides in male was higher than female. At the family level, the gut microbiota of the Brandt’s vole was dominated by Muribaculaceae (33.75%), Lachnospiraceae (25.99%), Prevotellaceae (6.90%) and Enterobacteriaceae (1.96%) (Fig. 2C). At the genus level, the dominant genus was *Lachnoclostridium* (2.25%), *Alistipes* (0.9%), and *Helicobacter* (0.6%) (Fig. 2D).

**Figure 3:**
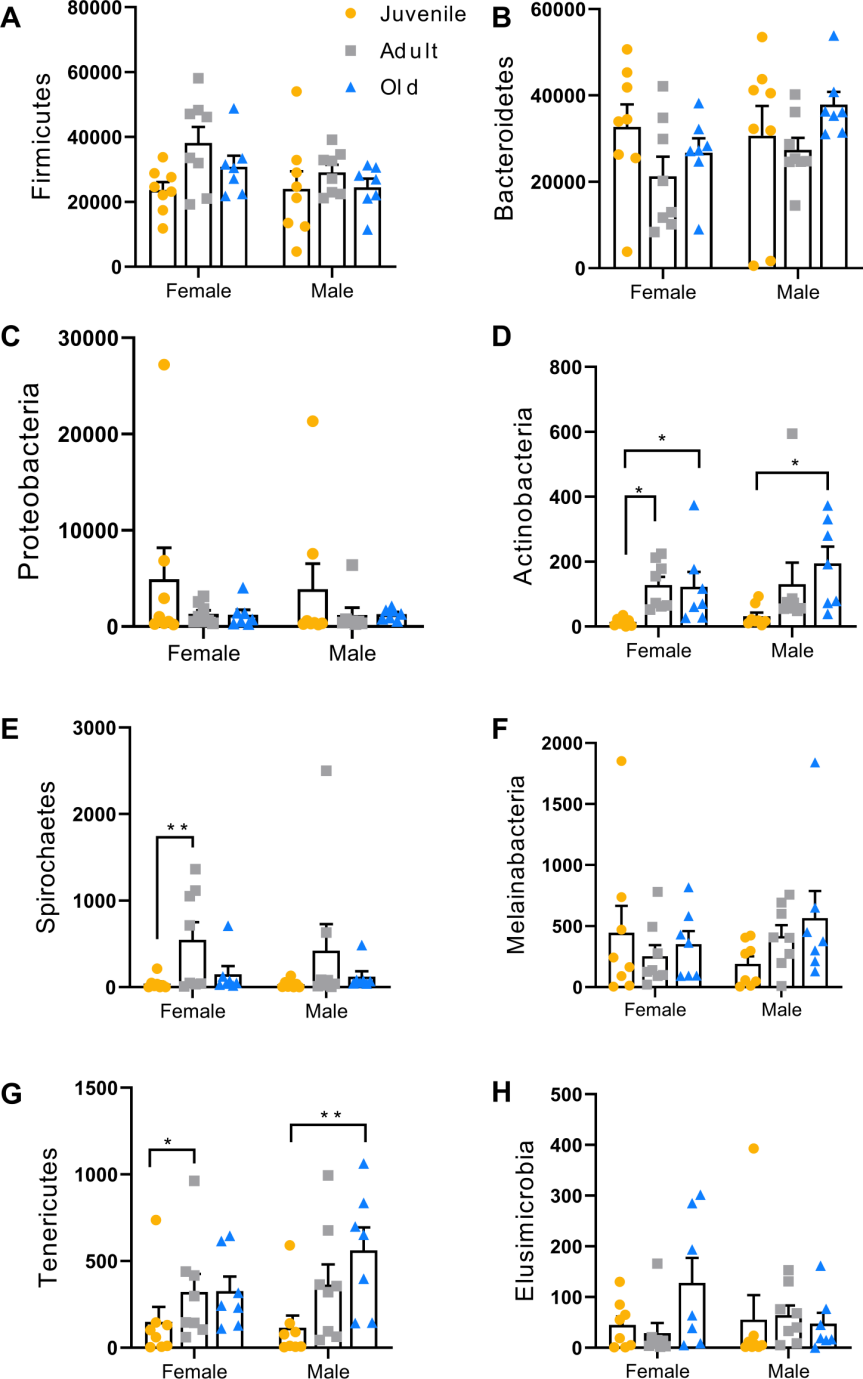
Absolute abundance of different phylum. (A) Firmicutes, (B) Bacteroidetes, (C) Proteobacteria, (D) Actinobacteria, (E) Spirochaetes, (F) Melainabacteria, (G) Tenericutes, and (H) Elusimicrobia in fecal microbiota community in different sex in young, adult and old groups. Data are means ± SEM (Female-juvenile: *n* = 7, Female-adult: *n* = 8, Female-old: *n* = 8; Male-juvenile: *n* = 7, Male-adult: *n* = 8, Male-old: *n* = 8). (Kruskal-Wallis, *p < 0.05).

**Figure 4:**
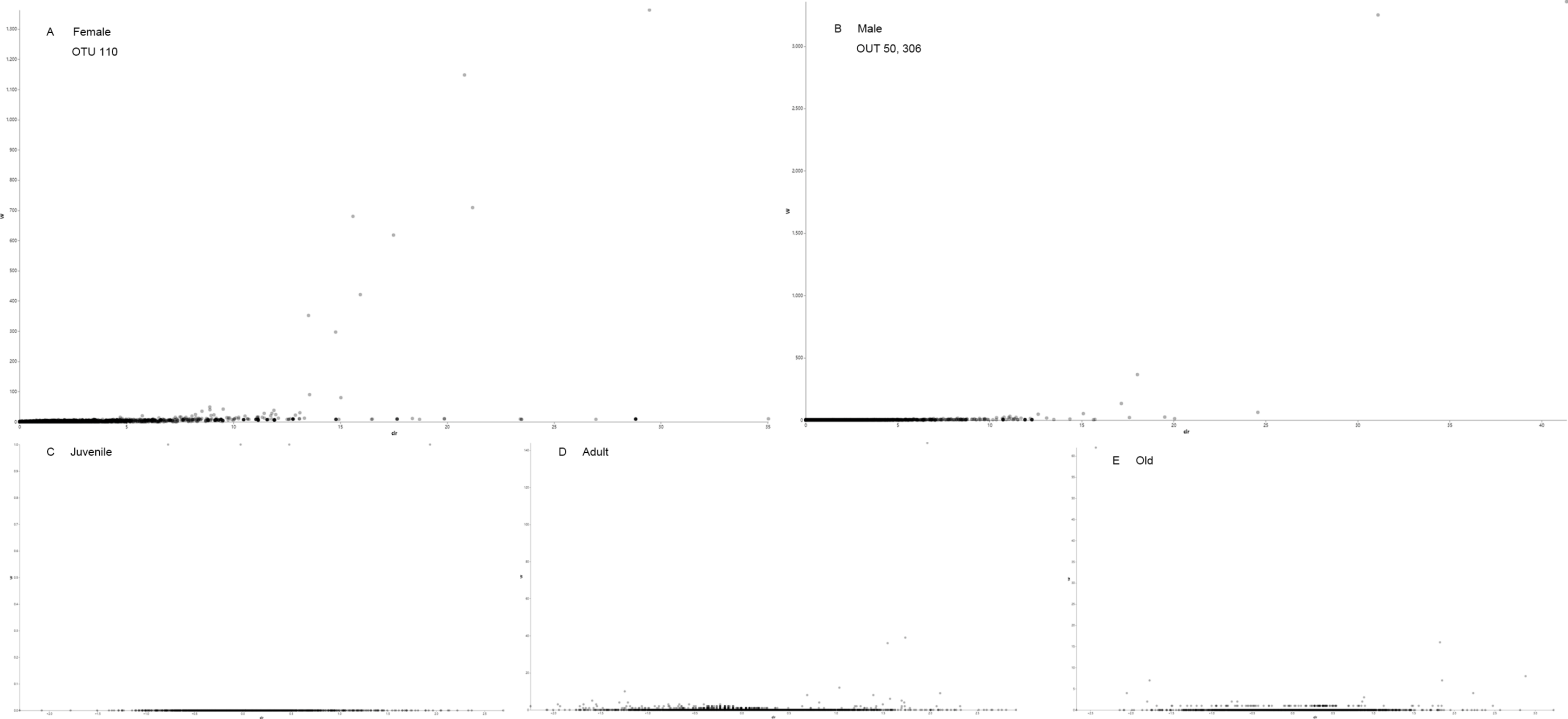
Results of ANCOM in different group. (A) Female, (B) Male, (C) juvenile, (D) Adult, (E) Old.

From Kruskal-Wallis at phylum level, we found that abundance of Firmicutes showed significant difference between six groups (Fig. 3A, *p* = 0.03), but it was no significantly different from the post-hoc; Actinobacteria, Tenericutes and Spirochaetes abundance showed significant difference between 6 groups (Fig. 3D, e.g., Actinobacteria: *p* < 0.001; Tenericutes: *p*  < 0.00; Spirochaetes: *p* = 0.013). In female voles, Actinobacteria (*p* = 0.03), Tenericutes (*p* = 0.03) and Spirochaetes (*p* = 0.009) in juvenile was lower than adult voles; also Actinobacteria (*p* = 0.012) in juvenile was lower than old voles. In male voles, Actinobacteria (*p* = 0.008) and Tenericutes (*p* = 0.007) in juveniles was lower than old voles (Fig. 3D, e,g). From ANCOM, we analyzed OTUs in different ages or sexes. The results showed that in female (Fig. 4A), there was only one OTU (OTU110) different between 3 ages. In male (Fig. 4B), there were two OTUs (OTU 50, OTU 306) different between 3 ages, while there was no difference between sexes in different age groups (Figs. 4C–4E).

**Figure 5:**
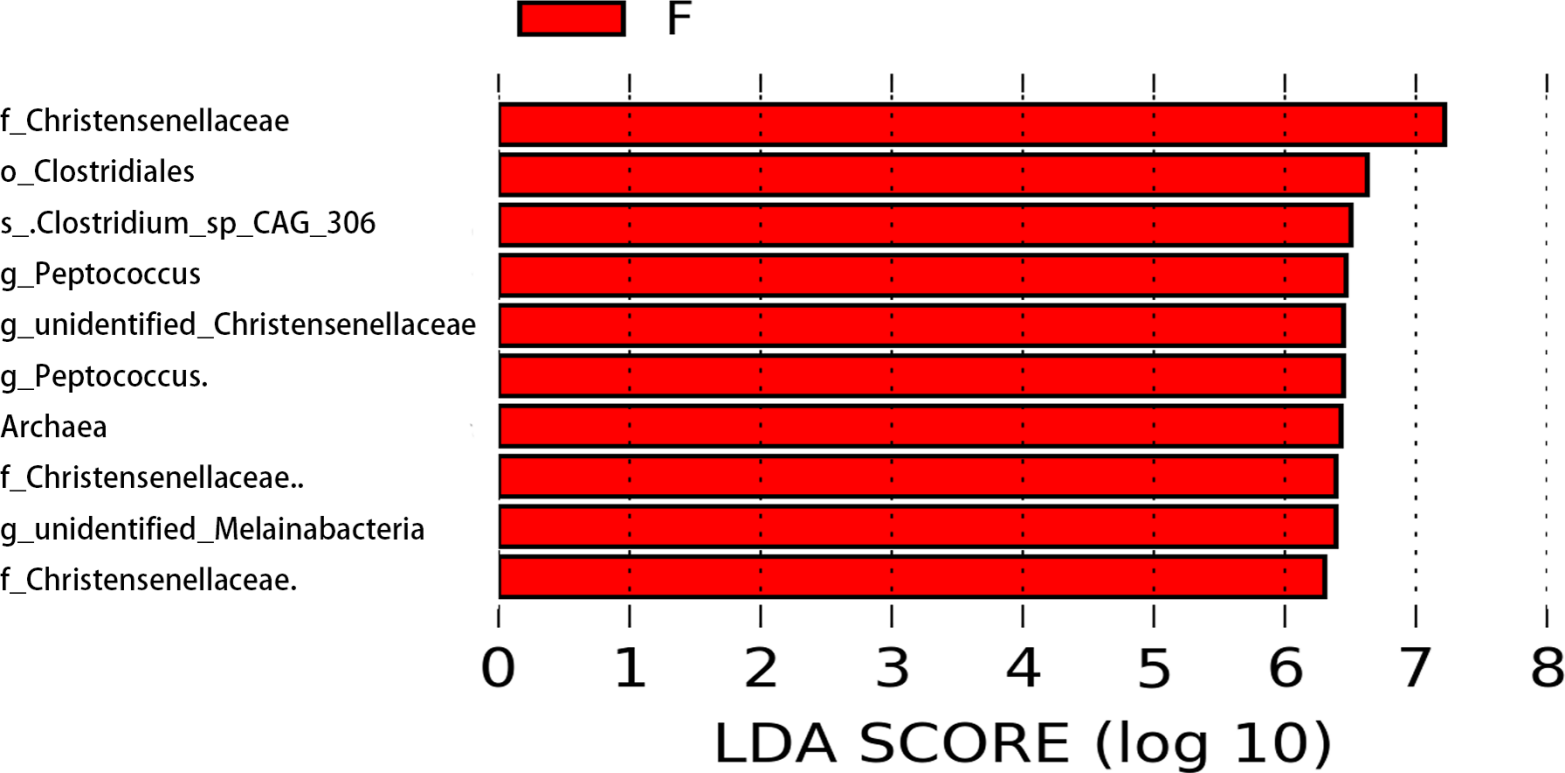
Differential bacterial taxonomy selected by LEfSe analysis with LDA score >2 in gut microbiota community between female and male in adult voles.

Through LEfSe analysis, we found that in adult, the abundances of one family *Christensenellaceae* (LDA score = 6.38, *p* = 0.026) and one genus *Peptococcus* (LDA score = 6.46, *p* = 0.027) of female were significantly higher than male voles (Fig. 5).

## Discussion

The gut microbiota of animals is a highly diverse ecosystem that varies drastically between individuals in response to environmental changes. It is vital to understand the factors affecting the diversity and composition of gut microbiota in the host at different life stages. In this study, we analyzed the associations of gut microbial composition and diversity with age and sex in wild Brandt’s voles. We found that the composition and structure of intestinal microbiota in voles were affected by sex and age in the same season.

### Associations of gut microbiota with age

Several researches have pointed a positive correlation between age and intestine microbiota alpha diversity from birth to adulthood (Hopkins, Sharp & Macfarlane, 2002). We found that there was no difference in microbial diversity between adult and old group in Brandt’s voles, which is consistent with studies in humans that there was no significant difference in alpha diversity of gut microbiota between middle-aged and 70-year-old people. However, in human studies, the alpha diversity of centenarians is higher than that of middle-aged people (Kato et al., 2017; Kong et al., 2016), likely due to the impacts of healthy aging, which is closely related to medical care and environment. Different from humans, the living environment of Brandt’s voles in the wild is very harsh. As the same time, our analyses indicated that the alpha diversity of fecal gut microbiota of Brandt’s voles was lowest in the juvenile group for both males or females, which were also consistent with some previous studies (e.g., Gilbert, 2015).

The current study showed that the most abundant phylum in the cecum of Brandt’s voles is Firmicutes and Bacteroidetes, which is consistent with other small mammals, such as rabbits (Velasco-Galilea et al., 2018), mice (Chevalier et al., 2015), and pikas (Li et al., 2018). Different from studies using laboratory animals (Liu et al., 2020), our results showed that the wild Brandt’s voles have more Proteobacteria phylum, especially in the juvenile group. This is associated with a complex environment and a high risk of infection. Some studies have shown that Proteobacteria, not Firmicutes and Bacteroidetes, can explain the variability of intestinal microbiota (Bradley & Pollard, 2017). Our results supported the hypothesis that wild voles harbor an unstable gut microbial community, characterized by an abundance of Proteobacteria. There was an association between a metabolic disorder and the expansion of Proteobacteria (Shin, Whon & Bae, 2015). In the juvenile group, instability of the microbial community has been interpreted as an impaired resistance to colonization. As the increase in age, the proportion of Proteobacteria decreased, and the stable microbial community gradually formed.

In our study, Brandt’s vole was dominated by *Muribaculaceae*, *Lachnospiraceae*, many members of these series are host to diverse genes encoding cellulases, hemicellulases, or oligosaccharide-degrading enzymes (Dai et al., 2015), which play critical functions in the metabolism of plant polysaccharides. *Lachnospiraceae* belongs to Clostridium of the phylum Firmicutes, and it can ferment glucose to produce formic acid, lactic acid, acetic acid. At the genus level, *Flavonifractor* was higher in female adults, which also belongs to the phylum Firmicutes and can synthesize short-chain fatty acids from plant polysaccharides (Zhang et al., 2020a; Zhang et al., 2020b; Zou et al., 2020; Mikami et al., 2020). The results of Bo et al. (2019) showed that the most abundant bacteria in the cecum of captive Brandt’s voles are Firmicutes and Bacteroides, accounting for about 91%, while the content of Proteobacteria is low. In contrast, the research results of grazing enclosures by Li et al. showed that Firmicutes had a significantly lower abundance and Bacteroidetes had a significantly higher abundance. Our results are similar to those of Li et al. (2019), the proportion of Proteobacteria in the juvenile and adult voles is relatively high, which may be related to their living environment in the wild.

In general, the function of the microbiota varies in different age groups, which matches the growth needs. We found abundance of some microbiota changed significantly with increase of age of voles, e.g., Firmicutes (*Flavonifractor*, *Oscillibacter*, *Anaerofilum*, *Blautia*, *Peptococcus*, *Acetatifactor*, *Butyricicoccus*, *Roseburia*, *Anaerovorax*, *Caproiciproducens*, *Lachnoclostridium*, *Ruminiclostridium*, *Intestinimonas*), Proteobacteria (*Desulfovibrio*, *Parasutterella*, *Tyzzerella*, *Helicobacter*), and Bacteroidetes (*Odoribacter*, *Butyricimonas*,). Many of them (e.g., *Flavonifractor*, *Enterorhabdus*, *Desulfovibrio*, *Elusimicrobium*, *Roseburia*, *Candidatus* _*Saccharimonas*, *Anaerovorax*, *Caproiciproducens*, *Lachnoclostridium*, *Ruminiclostridium*, *Intestinimonas*) play a key role in immunity regulation and intestinal health (Zhang et al., 2020a; Zhang et al., 2020b; Zhao, Jiang & Chu, 2020; Ward et al., 2017; Zhang et al., 2020a; Zhang et al., 2020b). However, *Unidentified_Clostridiales*, *Oscillibacter*, *Anaerofilum*, *Parasutterella*, *Tyzzerella*, *Blautia*, *Peptococcus*, *Mycoplasma*, *Helicobacter*, *Butyricimonas* have pathogenic and pro-inflammatory effects (Xu et al., 2020; Xue et al., 2020; Xie et al., 2020). The change in their abundance of these bacteria with age may be a potential reason for the shorter life span (around one year) of Brandt’s voles in the wild.

### Associations of gut microbiota with sex

Gut microbiome is closely related to host sex and is involved in the regulation of host metabolism and development, while its composition changes throughout the life-span of the host (Rizzetto et al., 2018). Several studies have reported variations in the diversity and composition of the gut microbiota between the sexes of mice (Markle et al., 2013). In our study, no variation in beta diversity were found between females and males in all age groups. We speculated that it was related to the small sample size or the large difference within the group. Especially in the juvenile group, the difference of beta diversity within the group is even higher than that between them and the adult or old group.

Some studies have shown sex differences in the gut microbiome of older mice, such as the decrease of Actinobacteria in males and the increase of Firmicutes in females (Ma et al., 2020). For example, Blautia and Bacteroides, which produce bile acids, are sex-dependent in regulating the health of both sexes. We found that in adult voles, the abundances of *Christensenellaceae* and *Peptococcus* were significantly higher in female than male voles. Differences in the microbial communities of male and female may be mediated by sex hormones, and differences in microbial community composition alter with age. For humans, early puberty and different sex hormones in women compared to men may lead to a more rapid diversification of the female gut microbiome (De et al., 2019).

## Conclusions

In summary, our study revealed the differences of gut microbiota between age and sex groups in wild animals. Different from previous studies using samples from laboratory animals, we found gut microbiota of wild voles was much influenced by their life-history reflected by their age and sex. In their gut microbiota, in order to adapt to the wild environment, there are many bacteria that help the host to resist infection. Future studies will be directed to identify functions of these unique microbiota in regulating physiological or behavioral processes of voles in different life stage or sexes.

## Additional Information and Declarations

### Data Availability

The following information was supplied regarding data availability: The raw sequence data are available at the National Center for Biotechnology Information (NCBI) Sequence Read Archive (SRA) with accession number PRJNA675310.
